# Barriers and facilitators of help-seeking among unemployed persons with mental health problems: a qualitative study

**DOI:** 10.1186/s12913-017-1997-6

**Published:** 2017-01-17

**Authors:** Tobias Staiger, Tamara Waldmann, Nicolas Rüsch, Silvia Krumm

**Affiliations:** Department of Psychiatry II, Ulm University and BKH Günzburg, Parkstraße 11, 89073 Ulm, Germany

**Keywords:** Mental health, Unemployment, Help-seeking, Service use, Stigma, Mental health literacy

## Abstract

**Background:**

Unemployed people with mental health problems often do not use mental health services and therefore do not benefit from available therapies. As unemployed individuals outside the healthcare system are a hard-to-reach group, barriers to and facilitators of mental health service use are poorly understood. The purpose of this study was to identify barriers to and facilitators of help-seeking and service use based on experiences of unemployed people with mental health problems.

**Methods:**

Fifteen qualitative semi-structured individual interviews were conducted with unemployed persons who reported mental health problems. Interview topics included individual experience with help-seeking and mental health service use with a focus on barriers and facilitators. Transcripts were analysed using qualitative content analysis and major themes were identified.

**Results:**

Participants reported being treated as “different” within their social environment as well as by health care professionals because of their mental health problems, which resulted in a lack of self-esteem and avoidance of help-seeking. Interviewees associated negative attributes with help-seeking such as helplessness and weakness. They equated psychiatric medication with illegal drugs and worried about the risk of addiction. However, social support and a desire for change on the other hand increased the motivation to search for help. Employment agency staff were mostly perceived as supportive by individuals seeking mental health services.

**Conclusions:**

Unemployed individuals with mental health problems faced barriers and facilitators when seeking help on three different levels: (1) mental health literacy; (2) stigma and discrimination; and (3) structures and conditions of health care. Awareness and attitudes of health care professionals concerning mental health issues should be improved. Stigmatisation of people with mental illnesses should be reduced in health care settings. Training for employment agency staff concerning mental health problems and services is recommended.

**Electronic supplementary material:**

The online version of this article (doi:10.1186/s12913-017-1997-6) contains supplementary material, which is available to authorized users.

## Background

### Unemployment, mental health and help-seeking

Mental health problems may be both a result of and a risk factor for unemployment [1]. Unemployed individuals have a higher risk of mental illness (odds ratios between 1.5 and 3.5) than employees [2, 3], and report higher levels of depressive symptoms and anxiety [4]. Health effects of unemployment depend on several factors, including education, income during unemployment and the duration of joblessness. The longer the unemployment, the higher the mental strain [5, 6]. However, unemployed people with mental health problems often choose not to use mental health services and therefore do not benefit from available psychopharmacological and psychosocial therapies [7]. The resulting treatment gap has harmful consequences for individuals, families and society, such as poor clinical outcomes, deterioration of health and longer unemployment. Higher rates of service use would decrease the illness-related burden of mental disorders [8] and greatly reduce the economic costs of mental illness [9].

Besides clinical symptoms, insight, accessibility and quality of services [10, 11], two additional factors influence the choice whether or not to seek help: knowledge about mental illness and available treatments [12] and stigma as a barrier to help-seeking [13, 14]. Both factors have the advantage of being potentially amenable to interventions. If people do not recognise mental illnesses or are not aware of the potential benefits of treatments, they are less likely to seek help [15].

### Stigma as a barrier to mental health care

The stigma of mental illness is common and describes a process involving stereotypes, prejudice and discrimination [16, 17]. Corrigan [13] reports that three factors influence help-seeking behaviours of people who might benefit from mental health treatment: public stigma (“all people with mental illness are dangerous”); self-stigma (“I have a mental illness, so I must be dangerous”) and structural stigma, which involves processes that represent macro level units rather than individuals, for example, waiting times for treatment as a consequence of limited funding for mental health services [13]. Stigma is an obstacle to seeking help in two equally pernicious ways [18, 19]. First, people with mental illness may avoid treatment in order not to be labelled “mentally ill” and to avoid public stigma as a negative consequence of the label; second, they can suffer from self-stigma and demoralisation, which undermines their motivation to seek help [20]. A systematic review concerning the impact of mental health-related stigma on help-seeking showed negative associations between stigma and help-seeking [14]. Furthermore, qualitative findings suggest a relationship between stigma and help-seeking based on the following five items [14]: (1) dissonance between a person’s preferred self-identity and common stereotypes about mental illness; (2) experience of negative consequences; (3) preference for non-disclosure; (4) stigma-related strategies used by individuals to enable help-seeking; and (5) stigma-related aspects of care that facilitate help-seeking. Treatment-related stigma and label avoidance are common barriers to help-seeking. In two recent studies participants perceived help-seeking as a sign of weakness or a failure to cope with their own problems (treatment-related stigma). Others put off help-seeking because they felt stigmatised by being identified and labelled as mentally ill (illness-related stigma) [21, 22].

Unemployed people with mental health problems might be affected by stigma associated with their unemployment and with their mental illness. The general public associates unemployment with incompetence [23]. O’Donnell et al. [24] tested the impact of anticipated social discrimination on psychological distress and somatic symptoms in a sample of unemployed people. Participants with higher anticipated discrimination also reported greater distress as well as more physical health problems. As a consequence, unemployed people with mental health problems might be reluctant to seek help in order not to be labelled “jobless”.

### Aims and research question

In order to develop adequate services and to increase help-seeking in this vulnerable group, there is a need to better understand barriers to and facilitators of service use among unemployed individuals. Therefore the purpose of this study was to identify barriers to and facilitators of help-seeking and service use based on experiences of unemployed people with mental health problems. Since most previous studies so far were quantitative and examined associations between barriers and help-seeking, we used a qualitative approach to identify barriers and facilitators associated with help-seeking and service use among unemployed people with mental health problems.

## Methods

### Instrument

Based on the literature and informal discussions with service users and relatives, we developed a semi-structured interview guide [25] with a series of questions on the following topics (Table 1): (i) dealing with unemployment and mental stress; (ii) stigma-related facilitators of and barriers to service use; (iii) knowledge-related facilitators and barriers; (iv) any other potential barriers and facilitators; (v) recommendations for optimising mental health care services. Although the interview guide had few specific questions on facilitators compared to questions on barriers, open questions allowed participants to describe barriers as well as facilitators.

**Table 1:** Interview guide

	Topic	Question
i	Dealing with unemployment and mental stress	How do you deal with your joblessness?
		How do family/friends react?
		How do you deal with your psychological stress?
		Where are you looking for support?
ii	Stigma-related facilitators of and barriers to service use	How is your mental health?
		Can you report negative experiences or unfair treatment by others because you have mental health problems?
		Have you experienced stigma or discrimination because you used or wanted to use mental health services? If yes, would you tell us about a situation, please?
iii	Knowledge-related facilitators and barriers	What do you call a ‘mental illness’?
		What do you know about mental health, stress and mental illnesses?
		What do you know about how to prevent a mental illness?
		What would you do to help someone you know if she or he had a mental illness?
		If you had a mental illness, where would you seek help?
		Where and how do you find information about mental illnesses?
iv	Any other potential barriers and facilitators	If you decided to seek professional help for mental distress, would there be any barriers to actually getting help?
v	Recommendations for optimising mental health care services	Are there kinds of help you would recommend in the case of a mental illness?

### Recruitment

We recruited a socio-demographically, clinically and unemployment-related diverse sample. By means of handouts and flyers, participants were recruited from employment agencies and social organisations between November 2014 and February 2015. The wording on the flyers/handouts was “Are you unemployed and suffer from worries, distress or mental health problems?”. Inclusion criteria were: unemployment, self-reported psychological distress, speaking German fluently, age between 18 and 63; exclusion criteria were: a current paid job with more than 14 h/week or earning more than €450/month.

### Data collection

We carried out individual in-depth interviews with 15 unemployed persons who reported mental health problems until we reached theoretical saturation [26]. The duration of the interviews was between 30 and 100 min. Interviews took place either at home, in a café or in the facilities of Ulm University.

### Analysis

Data were analysed by Qualitative Content Analysis [27] using the following steps: (i) Interviews were audio-recorded and transcribed verbatim; (ii) potential categories were defined, derived from the theoretical background and research questions; (iii) inductive codes were formulated based on the material; (iv) codes were collated into potential themes and themes were checked for consistency with coded extracts across the dataset, themes were refined and summarised into categories. The interviews were coded independently by two researchers (TW, TS) so that codings could be compared. Discordant coding was discussed with other researchers until consensus was reached. We used MAXQDA 11 (www.maxqda.com) for data analysis. Interview citations were translated from German into English after the analysis.

### Validation

To validate our findings, interview participants were invited to comment on and discuss the findings. Four interviewees participated in a focus group which took 100 min and was audio-recorded and transcribed verbatim. The transcript was analysed using both inductive (bottom-up) and deductive approaches (the latter based on the initial interview guide, outlined above, and the thematic coding framework that emerged from the individual interviews) [28]. The focus group covered participants’ views about identified themes (e.g. in terms of plausibility and comprehensive coverage of topics), cross-validating the findings from the individual interviews. The focus group confirmed the following findings and did not lead to changes in descriptions of the findings.

## Results

### Sampling characteristics

Participants differed in terms of age, gender, ethnicity, socio-economic status, education level, clinical history, family status, form of living and years of unemployment. Most participants were born in Germany (80%), median age was 55 (from 19 to 63), mean age was 48 years and about half were female (7♀ and 8♂). One third was single and about half (53%) divorced, 60% lived alone. About one in two had a university entrance diploma (47%). About one quarter reported that they had depression (27%), followed by bipolar, schizoaffective and anxiety disorders. About half of the interviewees did not specify their mental disorder. The length of the current episode of unemployment was between two months and 15 years.

Based on the research question, we generated the main categories of “barriers to help-seeking and service use” and “facilitators of help-seeking and service use” (Table 2). The analyses provided information about the formulated sub-categories of mental health literacy as well as stigma and discrimination related to help-seeking. These aspects were generated deductively based on previous research on stigma- and knowledge-related barriers. Further findings pointed to barriers and facilitators related to structures and conditions of health care, generated inductively from our data.

**Table 2:** Barriers and facilitators of help-seeking and service use

Main Category	Barriers	Facilitators
Mental Health Literacy	Fear of side effects of psychopharmacological treatment	Gaining knowledge as motivation factor for treatment
	Ineffective psychiatric help	Awareness and acceptance of illness
Stigma and discrimination – experiences	Perceived discrimination by mental health care professionals	Growing acceptance of mental health service use
	Stigma in the social environment	
	Internalised stereotypes associated with help-seeking	
Structures and conditions of health care	Miscommunication between therapist and patients	GP as facilitator and supporter
	GPs’ lack of interest in mental health problems	Positive relationship between patient and therapist
		The role of employment agency staff

### Categories of findings: Barriers of help-seeking

#### Low mental health literacy

This category summarises statements about knowledge concerning psychological distress and help-seeking which influenced help-seeking decisions. The findings indicated fears of an adverse impact of psychopharmacological treatment. Interviewees were worried about experiencing a personality change and therefore rejected psychopharmacological treatment. As one male interviewee put it:

I really refuse medication. I have just witnessed this among my acquaintances. There’s someone who has changed to total euphoria. That’s probably because he has downed the according amount of antidepressants. He has become the exact opposite (Participant 1, ♂, 55y).

Furthermore, participants equated psychiatric medication with illegal drugs. As a consequence, they perceived medical treatment to be harmful and worried about the risk of addiction (Participant 2, ♂, 63y). Other respondents described psychiatric help as ineffective and not suitable for solving individual problems. One person said: “Nobody can help me anyway. I do not have this qualification, I have not found a job and it’s pointless anyway” (Participant 3, ♀, 54y). The interviewee mentioned her unemployment status as the key problem and refused to seek help. But there seemed to be a need for support, as further passages referred to social withdrawal: “And for me it’s really hard leaving the house at all. On the bus occasionally I break into sweats. I don’t feel well among people anymore” (Participant 3, ♀, 54y).

#### Stigma and discrimination

The interviewees addressed experiences of unfair treatment due to mental illness. They reported discrimination by mental health professionals. Some respondents described experiences of stigma and physical abuse, for example, after a compulsory admission. One man said:

We were the scum of the earth for them. They were infamous especially for their mechanical restraints. Some of them really enjoyed using these restraints and one of them once confessed to me that he really liked doing so aggressively (Participant 4, ♂, 61y).

Others reported negative experiences within their social environment. A respondent described the reactions of family and neighbours to his mania, which made it harder to seek help in the future:

My wife got scared and called the police and the ambulance and they, of course, came and took me with them. As a consequence we lost our flat because a neighbour said she was scared of me and did not want to live close to a mentally ill person (Participant 4, ♂, 61y).

Other interviewees reported a lack of empathy among family and friends. One participant said: “They are normal down-to-earth people who say ‘Come on, it’s not that bad, don’t make such a fuss, everything will be alright’ and so on” (Participant 1, ♂, 55y). Furthermore, participants felt under pressure because they were not successful at university. This led to additional strains, which worsened the respondent’s situation:

I just feel bad because I feel that I disappoint them by, for example, not pulling off my studies. And so things get worse and worse because I feel I disappoint them and they will condemn me (Participant 5, ♂, 25y).

Further analysis showed that interviewees refused to seek help due to treatment-related stigma. One respondent (Mr. Schmidt) spoke of himself in the third person: “To say ‘Would you help me, please?’ Schmidt is not able to say that, Schmidt will not say that. Before he would say that, he’d rather shoot himself” (Participant 2, ♂, 63y). The interviewee associated help-seeking with specific attributes. He described that he felt helpless and injured, when seeking mental health services:

I don’t want to play the helpless and the mortally wounded. I don’t like that. That’s not me. I’m hanging in there, if necessary. And if I really cannot go on, there is another solution for me (Participant 2, ♂, 63y).

On the other hand participants felt a growing societal acceptance to disclose mental health problems and seek professional help. One respondent noted:

For a few years it’s been so in the open. As far as psychologists and psychiatrists are concerned, there’s no secret *(whispers) *‘I go to see a psychologist’. Back then things were different (Participant 6, ♀, 58y).

#### Structures and conditions of health care organisations

As well as barriers related to mental health literacy, participants reported structural barriers to psychiatric help. The interpersonal level in the communication between therapist and patient seemed to be key for successful service use. Therefore miscommunication was also mentioned as a barrier:

I said ‘Well, young man, I have not taken one step forward through the conversation we’ve just had. You have not let go anything at all, you have only listened to me very little. And then you cut in and tried twisting Schmidt’s words. You want to sell me something, that’s all’ (Participant 2, ♂, 63y).

The participant criticised that the therapist did not listen to him and therefore did not understand the patient’s needs. As a consequence the participant ended the psychotherapy. Another interviewee reported his experiences in a psychiatric hospital:

The female nurses didn’t care anyway, and the male nurses used to hide in the nurses’ station and all they did was dispense some medication. The doctors did not stand by us anyway. They rather questioned us but, eh, these were not really helpful, supportive conversations (Participant 4, ♂, 61y).

Further respondents criticised a lack of interest in mental health problems by general practitioners (GPs). An interviewee described the reaction of his GP:

I don’t know – he gave me the impression that mental health was not his area of expertise. Yeah – that he was rather focused on doctor’s stuff and disregarded mental health (Participant 7, ♂, 63y).

The restricted view of emotional stress led to a feeling of ‘not being taken seriously’ despite a need for help. As a result, a possible source of help was lost. One participant said:

In general, that’s the problem with my GP. He does not support me a lot. If I ask for a second opinion somewhere else he’d say ‘okay, go for it’, but he won’t ask for it himself. That’s not positive (Participant 7, ♂, 63y).

### Facilitators of help-seeking

#### Increased mental health literacy

Knowledge about psychological distress and help-seeking had a positive impact on mental health service use. Some people expressed their willingness to learn about mental disorders. Others indicated that illness awareness increased help-seeking. Since the interviewees’ reasons for help-seeking indicated an intrinsic motivation to seek information about possible health care services, gaining knowledge can be a motivational factor for dealing with one’s illness and to seek help: “…that I get some kind of input from the outside, maybe people giving me some advice so I can draw some kind of conclusions” (Participant 1, ♂, 55y). Due to better knowledge, some participants were more aware of their psychological functioning and had a better understanding of their symptoms: “[…] just because I read up on my illness I had this brought home to me: yeah, that’s what it is” (Participant 8, ♀, 22y). Moreover, an awareness of their illness seemed to have a positive impact on help-seeking and use of treatment. One participant explained his awareness of his poor mental health as the beginning of his help-seeking: “[…] it got worse and worse all the time, it didn’t get any better, and it finally dawned on me that I couldn’t make it on my own” (Participant 5, ♂, 25y).

Another motivation for help-seeking was the increased suffering combined with the inability to deal with it alone. One woman stated:

In the end there was no question I wouldn’t do it, because I really knew I wouldn’t make it on my own. I was getting worse and worse all the time and if I kept going like that, the emergency doctor would come and get me (Participant 9, ♀, 55y).

#### Enhancing structures and conditions of formal and informal help

Some participants described positive characteristics of GP and psychiatric care. Guidance by the GP was described and expected: “I would start with my GP, hoping he’d say to me ‘See this psychologist or psychiatrist, he’s good” (Participant 2, ♂, 63y). Others saw their GP as a source of emotional support: “As I said, I found it helpful that my doctor stood by me” (Participant 6, ♀, 58y). Regarding psychiatric-psychotherapeutic care, a positive relationship between patient and therapist seemed to be essential and was, for example, reflected by a welcoming attitude:

The staff made me feel very welcome. I felt totally integrated into the place. I had a mentor – I liked that, too. All the doctors were quite nice, I did not have any problems there at all (Participant 9, ♀, 55y).

Others referred to positive characteristics of therapists. Long-term support as well as a good personal relationship were perceived as crucial: “I already knew him as I was in his group therapy. He was a very nice and empathic person. He also helped me through my work life and when I developed test anxiety” (Participant 4, ♂, 61y). Additionally, sufficient time to talk with the therapist was considered important for mental health care: “These were one-on-one conversations. One-on-one conversations over a long period of time, we were not under time pressure at all and there were no requirements from the institution” (Participant 4, ♂, 61y).

Next to mental health care structures, employment agency staff were mentioned as important sources of help. For instance, after dropping out of a job programme, a person searched for help from her personal official:

I got kicked out of the programme. That’s why I had a talk with the employment agency official. At first he thought I just wasn’t interested and said ‘the notice of termination is fixed’. But then he realised that this affected me, that I wanted to be part of it. So he came up to me, started the conversation, listened to me, answered in a sensible way. I saw that he was interested, so I began to talk. That was good. I was more motivated and I felt better (Participant 10, ♀, 19y).

This interviewee described that, after she had left her job programme, the employment agency official first assumed she did not want to continue. Later the unemployment agency official developed interest in her situation. Due to his communicative abilities, the interviewee gained trust and started to talk about her mental health problems.

## Discussion

We set out to identify barriers to and facilitators of help-seeking among unemployed persons with mental health problems. We investigated factors related to the following three groups of themes: mental health literacy; experiences of stigma and discrimination; structures and conditions of health care. Concerning mental health literacy, our findings indicate that specific knowledge about treatment affected help-seeking and service use. Gaining knowledge about mental illnesses was one motivation to seek help. Awareness of one’s own situation and realising that it was not possible to deal with it alone also facilitated help-seeking. These results are in line with previous findings that people who recognise their mental health problems are more likely to seek professional help or psychopharmacological treatment [12, 19]. Findings highlighted worries about the adverse impact of psychopharmacological treatment and negative attitudes towards medication, which was perceived to be addictive and ineffective [29].

Other attitudes towards psychiatric treatment resulted from socio-economic conditions. In this study, unemployment and the resulting hopelessness were reasons not to seek help. Participants described psychiatric help as ineffective because joblessness was perceived as the main stressor. One possible explanation is based on three different phases of reactions to unemployment. After (i) a shock and, when all efforts fail, (ii) a pessimistic attitude concerning reemployment develops: “the individual becomes fatalistic and adapts himself to his new state but with a narrower scope. He now has (iii) a broken attitude” [30, 31]. Particularly the last phase of resignation might explain the lack of help-seeking. It appeared difficult, especially for the long-term unemployed, to motivate themselves to find treatment or a job. This may help to better understand the role of unemployment in non-help-seeking. Because of a “broken attitude” [30] interviewees tended to socially withdraw and therefore did not seek help. They stressed unemployment as their main issue and refused help-seeking for mental health problems.

With respect to mental health problems and help-seeking participants mentioned both public stigma and self-stigma [13]. Some reported being stigmatised by neighbours and therefore experienced discrimination based on stereotypes (“People with mental illness are dangerous”). In particular someone sought help and lost his flat because a neighbour did not want to live close to someone with a mental illness. These findings are consistent with a systematic review on the stigma associated with treatment for mental health problems [14]. Based on their analysis of qualitative studies, the authors emphasised the experience of negative consequences as a reason not to seek help.

Other participants reported a lack of empathy within their social environment. They did not feel they were taken seriously, especially by family members. This finding is supported by van der Sanden et al. [32] who described that family members of people with mental illness reported lower levels of perceived closeness to their ill relative than to extended family members. Moreover, participants felt discriminated by health care professionals and reported unfair treatment. This is consistent with previous research that, in general hospital settings, care and support for people with mental illness was poorer in comparison with patients without mental illness [33].

Further results showed that participants associated negative attributes with help-seeking. They felt helpless and vulnerable when they sought mental health services. This finding supports the hypothesis that negative societal images of those who seek psychological services may be internalised [13]. Help-seeking was seen as a sign of weakness or failure to cope with personal problems. Studies have shown that this type of treatment-related self-stigma seemed to be an important factor for help-seeking decisions. Vogel et al. [34] could show that perceived public stigma was positively related to self-stigma, that self-stigma was negatively associated with attitudes towards counselling and that these attitudes were positively associated with willingness to seek help for psychological concerns.

Our findings underline the role of structures and conditions of health care. In particular, communication between therapist and patient as well as a positive relationship facilitates help-seeking and service use. These results are in line with previous findings. Communication skills of GPs were shown to be an important factor affecting specific interventions for depression [35]. If GPs participated in a training programme of 20 h, depression treatment and patient outcomes improved significantly. If GPs were able to be an advisor in the context of depression treatment, as expected by our participants, patient outcomes could be improved. Other findings indicated a lack of interest in mental health problems on the part of GPs. The importance of this finding can be understood in the context of the Goldberg-Huxley model of the pathway to psychiatric care [36]. Its central thesis is that patients have to pass a series of filters before entering mental health services. The authors described the lack of recognition of mental illness by GPs as a possible obstacle to reaching mental health services. Furthermore, findings showed that participants refused to ask professional health care staff for help, but sought help in their social environment. Employment agency staff were seen as a first source to ask for help or advice.

Limitations of our study need to be considered. The inclusion of participants was not based on a detailed screening questionnaire, but on self-reported mental health problems and unemployment. Eighty per cent of the respondents were German citizens. Thus, it was not possible to examine differences in help-seeking behaviour between ethnic groups. Despite these limitations, our findings offer implications for future research and for interventions for improving help-seeking among unemployed persons with mental health problems. Awareness and attitudes of health care professionals concerning mental health issues should be improved, beginning with students of medicine and psychology. Training for employment agency staff on mental health problems and services would be helpful, for example, in terms of supported employment. This approach includes employment activities based on individual preferences and needs, integration of employment services into mental health services and personalised benefits planning [37]. Due to the fact that the concept of supported employment is not routinely implemented in the German health care system or the German Federal Employment Agency, policy changes and funding are needed to address this limitation. Since migration plays a major role in Europe, future studies should include more ethnically diverse samples to examine different coping styles and help-seeking needs in the case of mental health problems and unemployment.

## Conclusions

Unemployed individuals with mental health problems face barriers when seeking help, but also benefit from facilitators. These aspects can be divided into three different levels: (1) influence of low or increased mental health literacy: Participants fear an adverse impacts of psychopharmacological treatment and describe psychiatric help as ineffective. However, gaining knowledge on the other hand seems to be a motivational factor for treatment. Furthermore, awareness and acceptance of illness facilitate help-seeking. (2) Existence of stigma and discrimination: Although there seems to be more acceptance concerning help-seeking for mental health problems, stigma and discrimination by health care professionals as well as by friends and family continue. Participants see help-seeking as a sign of weakness and associate it with negative attributes such as helplessness. (3) Structures and conditions of health care: Miscommunication between therapist and patients as well as GPs’ neglect of mental health problems are barriers to help-seeking. In contrast, other respondents describe GPs as supportive and stress that a positive relationship between patient and therapist facilitates help-seeking. Finally, employment agency staff can assist with job searches as well as being a first place to turn to for help or advice.

## Additional file

Additional file 1: German responses. (DOCX 15 kb)

## Abbreviations

GP: General practitioner
