# Supplementary material for: Barriers and facilitators of help-seeking among unemployed persons with mental health problems: a qualitative study
Source: BMC Health Serv Res. 2017 Jan 17;17:39. doi: 10.1186/s12913-017-1997-6 (PMC5240360; doi:10.1186/s12913-017-1997-6)
Supplement: Additional file 1: — German responses. (DOCX 15 kb) [file 12913_2017_1997_MOESM1_ESM.docx]

**Table: German responses**

| **Participant** | **German response** |
| --- | --- |
| 1 | Wobei ich aber die Medikamentengeschichte auf jeden Fall ablehne. Weil ich hab das grad im Bekanntenkreis. Der hat sich von der Persönlichkeitsstruktur zu total euphorisch [verändert]. Wahrscheinlich, weil er dann die dementsprechenden Antidepressiva intus hatte. War der also komplett umgekehrt. |
|  | Das sind halt normale bodenständige Leute, die halt so dann sagen 'ah ist doch nicht so schlimm' und 'stell dich nicht so an' und 'das wird schon' und so. |
|  | Dass ich da eben von außen einen gewissen Input auch kriege, die mir vielleicht was raten können, ich selber was draus ziehen kann. |
| 2 | Zu sagen 'willst du mir helfen bitte?' Kann der Schmidt nicht, wird der Schmidt nicht machen. Vorher gibt er sich die Kugel. |
|  | Ich möcht net so dies vor allen Dingen net so hilflos und so tödlich verletzt spielen. Mag ich nicht, liegt mir nicht. Ich komm, wenn es sein muss, so durch und wenn es wirklich nimmer geht, dann gibt es für mich ne andere Lösung. |
|  | Ich sag 'also junger Mann, aus dem Gespräch, das wir jetzt hatten, komm ich kein Schritt weiter. Sie haben nichts losgelassen, sie haben nur ein bisschen zugehört. Und dann bei ein paar Punkten eingehakt und dann das, was Schmidt gesagt hat, versucht umzudrehen. Sie wollen mir was verkaufen, mehr war das nicht'. |
|  | Ich würde bei meinem Hausarzt anfangen, in der Hoffnung, dass der mir sagt 'geh mal zu dem Psychologen oder zu dem Psychiater, der ist gut'. |
| 3 | Kann mir sowieso kein Mensch helfen. Ich hab diese Ausbildung nicht, ich hab keinen Job bekommen und ist sowieso zwecklos. |
|  | Und für mich ist das wirklich ein Problem, überhaupt aus dem Haus zu gehen. Im Bus hab ich zeitweise Schweißausbrüche. Ich fühl mich nicht mehr unter Menschen wohl. |
| 4 | Wir waren auch für die letzter Dreck. Die waren berüchtigt, also grade wegen Fixierung. Haben ja manche also wirklich mit Vergnügen betrieben und einer hat mir mal gestanden, dass er das auch wirklich mit Aggression ganz gerne gemacht hat. |
|  | Da hat meine Frau Ängste entwickelt und hat Polizei und Krankenwagen bestellt und die kamen natürlich auch und haben mich mitgenommen. Und dann haben wir daraufhin die Wohnung verloren, weil eine Nachbarin gesagt hat, sie hat Angst vor mir und sie würde mit einem psychisch Kranken nicht zusammenwohnen wollen. |
|  | Die Schwestern hatten sowieso kein Interesse und die Pfleger, die haben sich da immer in der Kanzel verschanzt und bisschen Medikamente ausgegeben. Da hat man sowieso keine Unterstützung erfahren von den Ärzten. Das waren eher Verhöre aber ähm wirklich hilfreiche, helfende Gespräche waren das auch nicht. |
|  | Den kannte ich vorher schon. Ich war in einer Gruppentherapie bei ihm. Das war ein sehr netter, einfühlsamer Mensch. Der hat mich auch während des Berufs begleitet oder auch als ich damals so Examensängste entwickelt hab. |
|  | Das war halt ein individuelles Gespräch. Ein Einzelgespräch über längere Zeit, also mit keinem Zeitdruck und so weiter und keinen Vorgaben von der Institution her. |
| 5 | Ich fühl mich einfach schlecht, weil ich das Gefühl habe, dass ich die enttäusch dadurch, dass ich das Studium zum Beispiel nicht durchziehe. Und dadurch verschlimmert sich des auch, weil ich einfach das Gefühl hab, dass ich die enttäusch und dass die mich verurteilen. |
|  | […] dass es halt erst mal schlechter geworden ist die ganze Zeit und nicht besser und dass ich einfach gemerkt hab, dass es alleine nicht mehr geht. |
| 6 | Weil jetzt ist das alles offen seit ein paar Jahren. Mit dem Psychologen und dem Nervenarzt das ist jetzt nicht mehr so hinterrücks (flüstert) 'ich geh zum Psychologen'. Damals war es einfach noch nicht so. |
|  | Mir hat da auch wie gesagt damals geholfen, dass der Doktor zu mir gestanden hat. |
| 7 | Ich weiß nicht, er hat mir den Eindruck gemacht, dass er mit Psyche nicht so viel am Hut hat. Ja, dass er eigentlich mehr auf das Medizinische dann schaut und die Psyche so außer Acht lässt. |
|  | Es ist ja allgemein ein Problem mit meinem Hausarzt. Der steht mir da jetzt nicht so sehr bei. Wenn ich eine zweite Meinung irgendwo will, sagt er dann, dann soll ich sie mir holen, aber er macht es nicht. Das ist nicht so positiv. |
| 8 | […] und einfach dass man sich darüber informiert hat. Ja über die Krankheit, wo man gemerkt hat, oh das stimmt schon. |
| 9 | War für mich dann auch keine Frage mehr, dass ich das nicht mache, weil ich hab dann wirklich gemerkt, ich komm jetzt nicht mehr raus. Es wird immer schlimmer und wenn ich jetzt noch warte, dann kommt vielleicht noch der Notarzt und holt mich. |
|  | Man ist sehr nett aufgenommen worden vom Personal. Hast dich total integriert gefühlt. Hast einen Paten gehabt, das war auch ganz nett. Die Ärzte waren alle nett, also ich hab da eigentlich keine Probleme gehabt. |
| 10 | Ich bin aus dem Kurs halt rausgeflogen und deswegen hatte ich bei dem dann ein Gespräch und am Anfang dacht er, ich hab keine Lust und so. Hat er gesagt, 'dann bleibt’s bei der Kündigung' aber irgendwann hat er dann gemerkt, dass es mir schon also nahe gegangen ist, dass ich da halt gern wieder reinmöchte. Dann ist er halt auf mich zugekommen, hat angefangen, mit mir zu sprechen, hat mir auch gescheit zugehört, hat gescheite Antworten gebracht. Da hab ich halt gesehen, dass das auch ihn interessiert. Dann hab ich halt angefangen, zu reden. Und das fand ich gut. Dann hat ich schon mehr Motivation, ging es mir besser. |
